# Molecular Detection of Leptospiral DNA in Environmental Water on St. Kitts

**DOI:** 10.3390/ijerph110807953

**Published:** 2014-08-07

**Authors:** Julienne Rawlins, Alexandra Portanova, Ilana Zuckerman, Amanda Loftis, Pietro Ceccato, Arve Lee Willingham, Ashutosh Verma

**Affiliations:** 1 Ross University School of Veterinary Medicine, Basseterre, St. Kitts, West Indies; E-Mails: JRawlins@rossvet.edu.kn (J.R.); alexportanova@gmail.com (A.P.); IlanaZuckerman@students.rossu.edu (I.Z.); adloftis@gmail.com (A.L.); awillingham@rossvet.edu.kn (A.L.W.); 2 International Research Institute for Climate and Society, The Earth Institute, Columbia University, Palisades, NY 10964, USA; E-Mail: pceccato@iri.columbia.edu

**Keywords:** *Leptospira*, molecular detection, environmental transmission, leptospirosis

## Abstract

Leptospirosis is an important waterborne zoonotic disease caused by pathogenic *Leptospira*. The pathogen is maintained in a population due to chronic colonization and shedding from renal tubules of domestic and wild animals. Humans and other animals become infected when they come in contact with urine from infected animals, either directly or through urine-contaminated surface water. In this study, we screened environmental water on the island of St. Kitts by using a TaqMan based real time quantitative polymerase chain reaction (qPCR) targeting a pathogen specific leptospiral gene, *lipl32*. Our results indicate that around one-fifth of tested water sources have detectable leptospiral DNA.

## 1. Introduction

Leptospirosis is an important waterborne disease across the Caribbean islands that accounts for significant morbidity and mortality in animals and humans [1]. The spectrum of clinical presentations in human leptospirosis is very broad, ranging from a sub-clinical form to a potentially fatal syndrome involving multi-organ failure. Leptospirosis in dogs, cattle, horses and pigs is characterized by varied signs including renal and/or reproductive failure. Wild carrier animals, such as rodents and mongooses, play an important role in the transmission cycle and are often asymptomatic [2,3,4].

Leptospirosis is maintained in a population due to chronic kidney infection of a wide variety of domestic, peridomestic and wild reservoir mammals. Pathogenic leptospires live in the proximal renal tubules of these animals and are shed in their urine, contaminating surface water and soil, where leptospires can survive for weeks to months [5]. Exposure to contaminated environmental surface water is a frequent means of transmission to humans and healthy animals. Leptospirosis is considered an occupational threat to workers who are exposed to open water sources or animals, including veterinarians, farmers, abattoir workers, meat inspectors and rodent control workers. Leptospirosis outbreaks have been reported shortly after natural disasters such as flooding and hurricanes. Leptospirosis is also a recreational hazard for people who swim and wade in contaminated water [6,7].

In this study, we investigated leptospiral contamination of surface water on the island of St. Kitts*.* To that end, we screened surface water samples from various locations on the island by a real time quantitative PCR (qPCR) [8]. This assay targets *lipl32* gene that is present in all pathogenic leptospiral species but is absent in intermediate or non-pathogenic species of *Leptospira*.

## 2. Experimental Section

### 2.1. Collection of Water Samples

**Figure 1:**
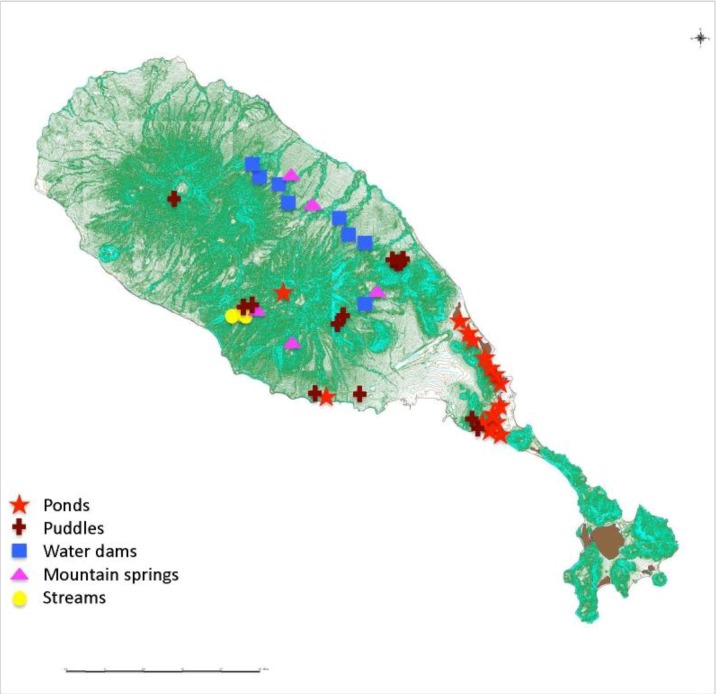
St. Kitts map showing sampling areas and types (https://www.google.com/maps).

Water samples were collected from 44 sites across the island (Figure 1), over a period of eight months. These sites included ponds, streams, mountain springs, water dams, and puddles. 

**Figure 2:**
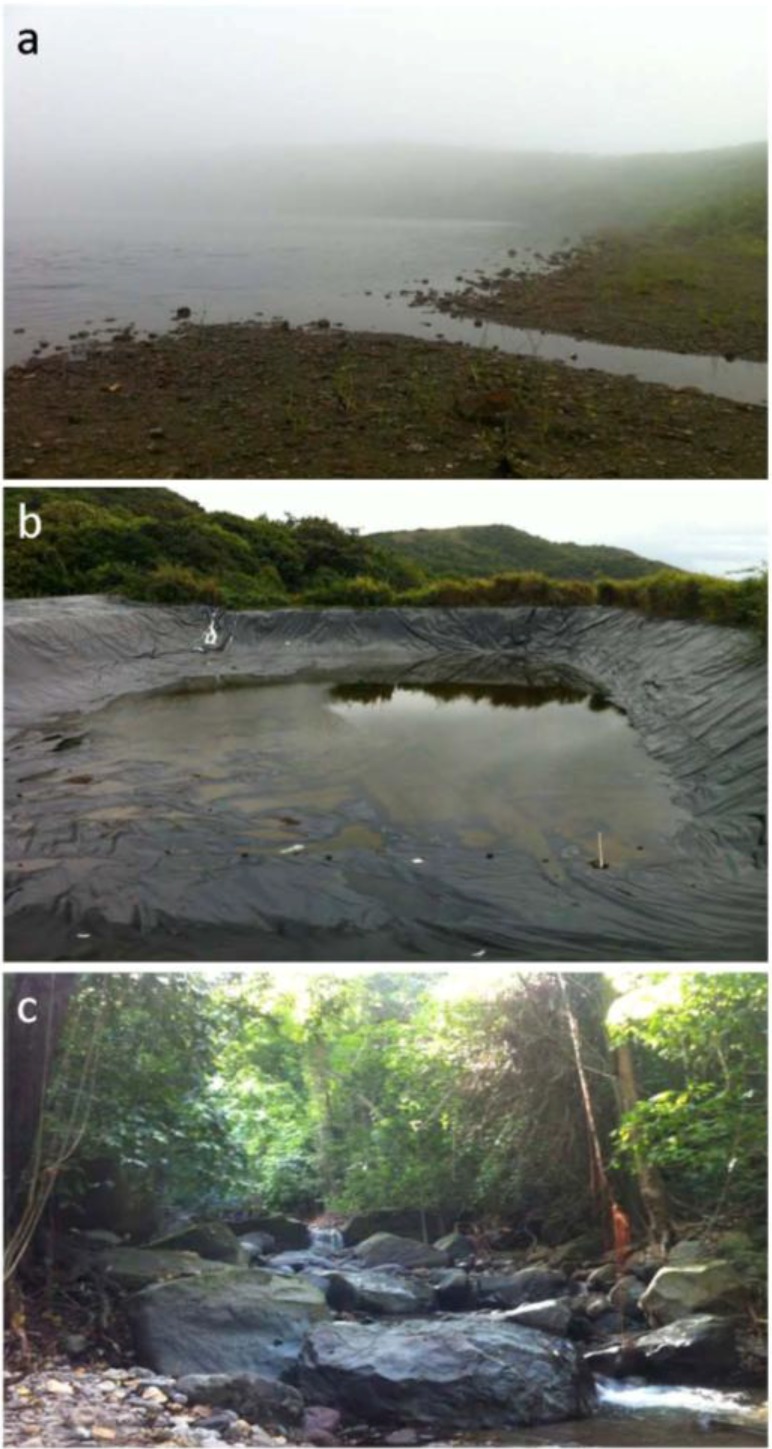
Sample collections sites: (**a**) a pond; (**b**) a water dam; and (**c**) a stream.

Mountain springs are sources of drinking water on the island. Water dams are 110 feet long, 100 feet wide and 12 feet deep reservoirs located in the mountains to collect rainwater that is used by farmers for irrigation (Figure 2). For qPCR, 300 mL water was collected from each site. Each sample received a unique number and was stored at −20 °C, not more than 48 h, until processed. Additional 300 mL water samples were collected from eight water dams and five mountain springs for culturing of leptospires. Culturing from other samples was not attempted.

### 2.2. Culturing of Leptospira *spp*.

For culturing, 300 mL of water samples collected from water dams and mountain springs were immediately centrifuged at 3000 g for 30 min at 6 °C and pellets were resuspended in Polysorbate-80 bovine serum albumin medium (NVSL) with 0.01% fluorouracil and incubated at 30 °C for 4 weeks. Each culture sample was weekly checked under the darkfield microscope for growth and contamination.

### 2.3. DNA Extraction

DNA was extracted from water samples using DNeasy Blood and Tissue kit (Qiagen, Valencia, CA, USA). Water samples were centrifuged as described above and pellets were processed following manufacturer’s instructions with some modifications [9].

*Leptospira interrogans* serovar Pomona was grown in Polysorbate-80 bovine serum albumin medium (NVSL) at 30 °C, and genomic DNA isolated, and quantified as previously described [10]. Based on the genome size of *L. interrogans* (4.659 Mb), genome equivalents were calculated as described [11]. *L. interrogans* DNA concentration of 550 μg/mL was equivalent to ~1.13 × 10^11^ genome units/mL [11].

### 2.4. Quantitative Polymerase Chain Reaction (qPCR)

We used a TaqMan based quantitative PCR (qPCR) to target a 242 bp region of leptospiral *lipl32* gene, as previously described [8]. The assay was performed in a MicroAmp Fast Optical 96-well reaction plate (Applied Biosystems, Foster City, CA, USA). Each plate contained DNA equivalent to 10^5^, 10^4^, 10^3^, 10^2^, 10, 1, 0.1 and 0.01 leptospiral genome units. Each column, except positive control columns, had a no-template control. Each reaction was performed in a 25 μL final volume, using 5 μL of extracted DNA, 500 nM of LipL32-45F (forward primer; 5'- AAGCATTACCG CTTGTGGTG-3'), 500 nM of LipL32-286R (reverse primer; 5'-GAACTCCCATTTCAGCGATT-3') and 100 nM of LipL32-189P (probe; FAM-5'-AAAGCCAGGACAAGCGCCG-3'-BHQ1) [8]. The assay was performed on a ABI 7500 (Applied Biosystems) using Platinum Quantitative PCR SuperMix-UDG (Invitrogen, Carlsbad, CA, USA) and thermal conditions of a holding stage of 95 °C for 20 s, and 40 cycles of 95 °C for 3 s and 60 °C for 30 s.

## 3. Results and Discussion

**Figure 3:**
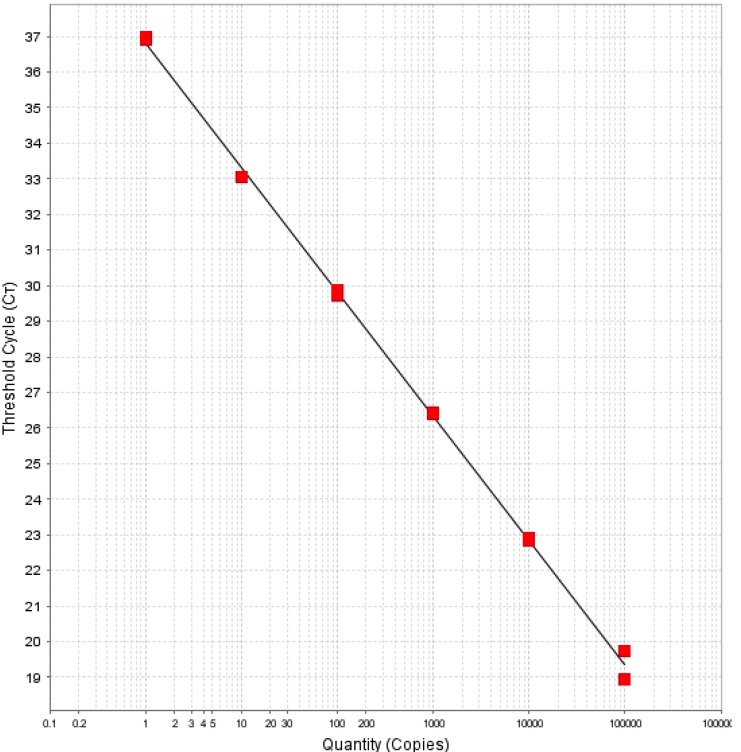
Standard curve obtained with 10^5^, 10^4^, 10^3^, 10^2^, 10, 1, 0.1 and 0.01 genome units of *L. interrogans* serovar Pomona. The slope of regression line between threshold cycle and leptospiral DNA standards is −3.485, and R^2^ is 0.999.

This qPCR method targets a gene, *lipl32*, which encodes for a 32 kDa membrane protein of pathogenic leptospires. *lipl32* is fairly conserved among pathogenic species but is absent in non-pathogenic and intermediate leptospiral species [8]. So, this assay does not detect saprophytic leptospires that may be present in the environmental samples. The assay was previously developed for the detection of leptospiral DNA in clinical samples [8]. In those studies it was found to be a robust assay with high sensitivity and specificity. We adapted this assay for detecting leptospiral DNA in environmental water. Three hundred milliliter of water sample, from each of 44 sites on the island, was collected (Figure 1). The DNA extracted from water samples was analyzed by the qPCR assay. For analysis, a threshold of 100,000 and baseline between 5 and 12 cycles were used. Experiments that did not meet the standard curve criteria of a slope between −3.33 and −3.60, and R^2^ value of more than 0.97 were invalidated. Duplicate samples that had Ct <40 were considered positive. Samples that were undetectable or had a Ct of >40 were considered negative. In addition, a run was considered valid only if all ten no-template controls were negative. Figure 3 shows a standard curve obtained with 10^5^, 10^4^, 10^3^, 10^2^, 10, 1, 0.1 and 0.01 genome units of *L. interrogans* serovar Pomona.

Table 1 summarizes our qPCR results. A total of 15 samples were collected from ponds and three samples were collected from streams. None of these samples were positive by our method. Out of eight water dam sites, five (62.5%) were positive for leptospiral DNA. Two out of five mountain springs were also positive. Only one out of 13 puddle sites was positive. The Ct values of positive samples ranged between 35.2 and 36.7, which is equivalent to 56 to 20.6 genome units per 300 mL of water sample.

The infectious dose of leptospires in humans is not known but incidents of infection acquired through recreational water exposure suggest a low dose [12,13,14,15,16].

**Table 1:** Quantitative PCR results for pathogenic *Leptospira* spp. in environmental water samples.

Source Type	Number of Samples Collected	Number Positive
Ponds	15	0
Puddles	13	1
Water dams	8	5
Mountain springs	5	2
Streams	3	0

The degree of environmental contamination depends on several factors including urine volume, frequency, concentration of leptospires in the urine, animal access to water sites, and water or soil type, temperature, and movement of water. Our results indicate that water dams provide conditions that increase chance of contamination by carrier animals and subsequent survival of the organism. These dams are open, large pits (110 × 100 × 12 feet) lined with waterproof sheets (Figure 2b) to collect rainwater for use in irrigation. Since the dams are not covered, they are easily accessible to rodents, mongooses, monkeys and other animals living in the mountains. Rodents and mongooses are asymptomatic reservoirs of the disease in different parts of the world and potentially a source of contamination of these sites. Mountain spring collection points are similarly accessible to wild reservoir animals. Most of the ponds are also accessible to mongooses and rats, albeit to a lesser degree, but were negative for leptospiral DNA. Although not tested, these stagnant water bodies may have very high concentrations of salt or other inhibitory factors for leptospiral growth.

Our attempts to culture leptospires from water dam and mountain spring samples were not successful because of contamination by other, less fastidious, bacteria. The TaqMan based quantitative PCR method used in this study is a useful tool for detecting leptospires in environmental water. It is a very sensitive assay that is specific for detecting pathogenic leptospires. In our hands, the detection limit of this assay is 1 genome equivalent. Because PCR assays detect DNA, this assay is also limited in that it cannot differentiate between dead and live bacteria; however, the half-life of free DNA is short, and the possibility of free DNA in these samples is extremely low [9,17].

Under favorable environmental conditions, pathogenic leptospires can survive in water and soil for prolonged periods. Though our study is primarily a point prevalence assessment, it provides important information on environmental contamination by leptospires on this small Caribbean island. The tropical climate of the island is ideal for survival of the pathogen in the environment. It is likely that mongooses, rats and monkeys contaminate water and soil and maintain circulation of *Leptospira* in the environment. Humans and other accidental hosts get infected through exposure to contaminated water. Mongooses and other wildlife species were found to be reservoirs of *Leptospira* in Grenada and Trinidad [18], but no similar study has been done to address that question on St. Kitts. Two recent studies have assessed the seroprevalence of leptospirosis among people on St. Kitts but no information is available on its prevalence in animals, reservoir hosts, or transmission of the disease [19,20]. Changes in water quality and quantity due to variations in precipitation and temperature might also affect the rate of transmission. Future studies will focus on understanding effects of environmental variables on transmission by using remotely sensed data from different satellites (*i.e*., ISERV on-board the International Space Station and LANDSAT). Information about the circulating serovars, their maintenance hosts and changes in the climatic and environmental conditions will be essential for understanding the epidemiology of leptospirosis on this island.

## 4. Conclusions

In this study we used a quantitative PCR assay to detect leptospiral DNA in surface water on St. Kitts. We found that around 18% of the sites had detectable levels of leptospiral DNA, but this contamination was mainly seen in water dams and other water collection points in the mountains. Water from these contaminated sites is used either for irrigation or drinking; thus, suitable measures should be taken to minimize animal access and educate people about the potential hazards of exposure to *Leptospira*-contaminated water. Stakeholder awareness of this evidence will be essential for enabling routine surveillance and control activities aimed at better understanding and reducing the risk of human and animal infection on the island.
